# Supplementary material for: End Sequence Analysis Toolkit (ESAT) expands the extractable information from single-cell RNA-seq data
Source: Genome Res. 2016 Oct;26(10):1397–410. doi: 10.1101/gr.207902.116 (PMC5052061; doi:10.1101/gr.207902.116)
Supplement: Supplemental Material [file supp_gr.207902.116_Supp_Materials.tar.gz › ESAT-esat_v0.1/doc/html/classbroad_1_1core_1_1siphy_1_1_tree_scaler.html]

ESAT: broad.core.siphy.TreeScaler Class Reference


ESAT


- Main Page
- Related Pages
- Packages
- Classes
- Files

- Class List
- Class Index
- Class Hierarchy
- Class Members

All Classes Namespaces Files Functions Variables Enumerator Pages

- broad
- core
- siphy
- TreeScaler

Public Member Functions |
Static Public Member Functions |
Static Public Attributes |
List of all members

broad.core.siphy.TreeScaler Class Reference

Collaboration diagram for broad.core.siphy.TreeScaler:

[legend]

|  |  |
| --- | --- |
| Public Member Functions | |
|  | TreeScaler () |
|  | |
|  | TreeScaler (Annotation region, File modelFile, String alnFile, String alnFileFormat) throws Exception |
|  | |
| void | setAlignment (String alnFile, String alnFileFormat) throws Exception |
|  | |
| void | encodeAsMatrix () |
|  | |
| double | getLogLikelihood (double omega, Phylogeny alnTree, int startPosition, int window) |
|  | |
| PiFit | fitPI (Phylogeny alnTree, int i) |
|  | |
| List< BED > | slidePWM (PositionWeightMatrix pwm, double minSeedScore, short[] encodedRef, List< int[]> ungappedChunks, int numPermutations) |
|  | |
| List< BED > | slidePWM (PositionWeightMatrix pwm, double minSeedScore, int numPermutations) |
|  | |
| OmegaFit | scaleRegion (List< String > ignoreList, GenomicAnnotation region) |
|  | |
| OmegaFit | scaleRegion (Annotation region) |
|  | |
| void | scaleTree (int window, BufferedWriter bw, List< String > ignoreList, int overlap) throws IOException |
|  | |
| ArrayList< OmegaFit > | scaleTree (int window, List< String > ignoreList, int overlap) throws IOException |
|  | |
| void | scaleTreeWithSampling (int window, BufferedWriter bw, List< String > ignoreList, int numberToAverage) throws IOException |
|  | |
| void | setAlignment (MultipleAlignment alignment) |
|  | |
| double | getTotalDistanceFromNode (PhylogenyNode n) |
|  | |
| void | setNeutralModel (File modelFile) throws IOException, ParseException |
|  | |
| double | getMinimumTreeLength () |
|  | |
| void | setMinimumTreeLength (double minimumTreeLength) |
|  | |
| EvolutionaryModel | getModel () |
|  | |
| void | setModel (EvolutionaryModel model) |
|  | |

|  |  |
| --- | --- |
| Static Public Member Functions | |
| static void | main (String[] args) throws Exception |
|  | |

|  |  |
| --- | --- |
| Static Public Attributes | |
| static final String | USAGE |
|  | |

## Constructor & Destructor Documentation

|  |  |  |  |  |
| --- | --- | --- | --- | --- |
| broad.core.siphy.TreeScaler.TreeScaler | ( |  | ) |  |

Here is the caller graph for this function:

|  |  |  |  |
| --- | --- | --- | --- |
| broad.core.siphy.TreeScaler.TreeScaler | ( | Annotation | *region*, |
|  |  | File | *modelFile*, |
|  |  | String | *alnFile*, |
|  |  | String | *alnFileFormat* |
|  | ) |  | throws Exception |

Here is the call graph for this function:

## Member Function Documentation

|  |  |  |  |  |
| --- | --- | --- | --- | --- |
| void broad.core.siphy.TreeScaler.encodeAsMatrix | ( |  | ) |  |

Here is the caller graph for this function:

|  |  |  |  |
| --- | --- | --- | --- |
| PiFit broad.core.siphy.TreeScaler.fitPI | ( | Phylogeny | *alnTree*, |
|  |  | int | *i* |
|  | ) |  |  |

Here is the call graph for this function:

|  |  |  |  |
| --- | --- | --- | --- |
| double broad.core.siphy.TreeScaler.getLogLikelihood | ( | double | *omega*, |
|  |  | Phylogeny | *alnTree*, |
|  |  | int | *startPosition*, |
|  |  | int | *window* |
|  | ) |  |  |

Here is the call graph for this function:

Here is the caller graph for this function:

|  |  |  |  |  |
| --- | --- | --- | --- | --- |
| double broad.core.siphy.TreeScaler.getMinimumTreeLength | ( |  | ) |  |

|  |  |  |  |  |
| --- | --- | --- | --- | --- |
| EvolutionaryModel broad.core.siphy.TreeScaler.getModel | ( |  | ) |  |

Here is the caller graph for this function:

|  |  |  |  |  |  |
| --- | --- | --- | --- | --- | --- |
| double broad.core.siphy.TreeScaler.getTotalDistanceFromNode | ( | PhylogenyNode | *n* | ) |  |

Here is the caller graph for this function:

|  |  |  |  |  |  |
| --- | --- | --- | --- | --- | --- |
| static void broad.core.siphy.TreeScaler.main | ( | String[] | *args* | ) | throws Exception |

static

Here is the call graph for this function:

|  |  |  |  |
| --- | --- | --- | --- |
| OmegaFit broad.core.siphy.TreeScaler.scaleRegion | ( | List< String > | *ignoreList*, |
|  |  | GenomicAnnotation | *region* |
|  | ) |  |  |

Here is the call graph for this function:

|  |  |  |  |  |  |
| --- | --- | --- | --- | --- | --- |
| OmegaFit broad.core.siphy.TreeScaler.scaleRegion | ( | Annotation | *region* | ) |  |

Here is the call graph for this function:

|  |  |  |  |
| --- | --- | --- | --- |
| void broad.core.siphy.TreeScaler.scaleTree | ( | int | *window*, |
|  |  | BufferedWriter | *bw*, |
|  |  | List< String > | *ignoreList*, |
|  |  | int | *overlap* |
|  | ) |  | throws IOException |

Here is the call graph for this function:

|  |  |  |  |
| --- | --- | --- | --- |
| ArrayList<OmegaFit> broad.core.siphy.TreeScaler.scaleTree | ( | int | *window*, |
|  |  | List< String > | *ignoreList*, |
|  |  | int | *overlap* |
|  | ) |  | throws IOException |

Here is the call graph for this function:

|  |  |  |  |
| --- | --- | --- | --- |
| void broad.core.siphy.TreeScaler.scaleTreeWithSampling | ( | int | *window*, |
|  |  | BufferedWriter | *bw*, |
|  |  | List< String > | *ignoreList*, |
|  |  | int | *numberToAverage* |
|  | ) |  | throws IOException |

Here is the call graph for this function:

|  |  |  |  |
| --- | --- | --- | --- |
| void broad.core.siphy.TreeScaler.setAlignment | ( | String | *alnFile*, |
|  |  | String | *alnFileFormat* |
|  | ) |  | throws Exception |

Here is the caller graph for this function:

|  |  |  |  |  |  |
| --- | --- | --- | --- | --- | --- |
| void broad.core.siphy.TreeScaler.setAlignment | ( | MultipleAlignment | *alignment* | ) |  |

|  |  |  |  |  |  |
| --- | --- | --- | --- | --- | --- |
| void broad.core.siphy.TreeScaler.setMinimumTreeLength | ( | double | *minimumTreeLength* | ) |  |

|  |  |  |  |  |  |
| --- | --- | --- | --- | --- | --- |
| void broad.core.siphy.TreeScaler.setModel | ( | EvolutionaryModel | *model* | ) |  |

|  |  |  |  |  |  |
| --- | --- | --- | --- | --- | --- |
| void broad.core.siphy.TreeScaler.setNeutralModel | ( | File | *modelFile* | ) | throws IOException, ParseException |

Here is the caller graph for this function:

|  |  |  |  |
| --- | --- | --- | --- |
| List<BED> broad.core.siphy.TreeScaler.slidePWM | ( | PositionWeightMatrix | *pwm*, |
|  |  | double | *minSeedScore*, |
|  |  | short[] | *encodedRef*, |
|  |  | List< int[]> | *ungappedChunks*, |
|  |  | int | *numPermutations* |
|  | ) |  |  |

Here is the call graph for this function:

Here is the caller graph for this function:

|  |  |  |  |
| --- | --- | --- | --- |
| List<BED> broad.core.siphy.TreeScaler.slidePWM | ( | PositionWeightMatrix | *pwm*, |
|  |  | double | *minSeedScore*, |
|  |  | int | *numPermutations* |
|  | ) |  |  |

Here is the call graph for this function:

## Member Data Documentation

final String broad.core.siphy.TreeScaler.USAGE

static

---

The documentation for this class was generated from the following file:

- java/broad/core/siphy/TreeScaler.java


---

Generated on Fri Aug 15 2014 15:10:57 for ESAT by  

 1.8.7
